# The DHAV-1 protein VP1 interacts with PI3KC3 to induce autophagy through the PI3KC3 complex

**DOI:** 10.1186/s13567-022-01081-6

**Published:** 2022-08-17

**Authors:** Juan Li, Mingshu Wang, Shan Zhou, Anchun Cheng, Xuming Ou, Di Sun, Ying Wu, Qiao Yang, Qun Gao, Juan Huang, Bin Tian, Sai Mao, Shaqiu Zhang, Xinxin Zhao, Renyong Jia, Mafeng Liu, Dekang Zhu, Shun Chen, Yunya Liu, Yanling Yu, Ling Zhang, Leichang Pan

**Affiliations:** 1 Institute of Preventive Veterinary Medicine, Sichuan Agricultural University, Chengdu, 611130 China; 2 Key Laboratory of Animal Disease and Human Health of Sichuan Province, Sichuan Agricultural University, Chengdu, 611130 China; 3 Avian Disease Research Center, College of Veterinary Medicine, Sichuan Agricultural University, Chengdu, 611130 China

**Keywords:** Duck hepatitis A virus type 1, VP1, autophagy, PI3KC3, Beclin1

## Abstract

Duck hepatitis A virus type 1 (DHAV-1) is one of the main pathogens responsible for death in ducklings. Autophagy is a catabolic process that maintains cellular homeostasis, and the PI3KC3 protein plays an important role in the initiation of autophagy. DHAV-1 infection induces autophagy in duck embryo fibroblasts (DEFs) but the molecular mechanism between it and autophagy has not been reported. First, we determined that DHAV-1 infection induces autophagy in DEFs and that autophagy induction is dependent on the integrity of viral proteins by infecting DEFs with UV-inactivated or heat-inactivated DHAV-1. Then, in experiments using the pharmacological autophagy inducer rapamycin and the autophagy inhibitor chloroquine, autophagy inhibition was shown to reduce intracellular and extracellular DHAV-1 genome copies and viral titres. These results suggest that autophagy activated by DHAV-1 infection in DEFs affects DHAV-1 proliferation and extracellular release. Next, we screened the autophagy-inducing effects of the DHAV-1 structural proteins VP0, VP3, and VP1 and found that all DHAV-1 structural proteins could induce autophagy in DEFs but not the full autophagic flux. Finally, we found that VP1 promotes protein expression of PI3KC3 and Beclin1 by western blot experiments and that VP1 interacts with PI3KC3 by co-immunoprecipitation experiments; moreover, 3-MA-induced knockdown of PI3KC3 inhibited VP1 protein-induced autophagy in DEFs. In conclusion, the DHAV-1 structural protein VP1 regulates the PI3KC3 complex by interacting with PI3KC3 to induce autophagy in DEFs.

## Introduction

Duck hepatitis A virus type 1 (DHAV-1), *Avihepatovirus* genus, *Picornaviridae* family, is one of the most common highly lethal hepatitis pathogens in ducklings [1–3]. The genome of DHAV-1 is a single, positive-stranded RNA and contains an open reading frame (ORF) that encodes an inactive precursor protein [4]. After multiple cleavage steps, the precursor protein produces the structural protein P1 region (capsid protein VP0, VP1 and VP3) and the nonstructural proteins P2 region (2A, 2B and 2C proteins) and P3 region (3A, 3B, 3C and 3D proteins) [5–10].

Autophagy is an evolutionarily conserved pathway involved in cellular homeostasis and organelle renewal in eukaryotes [11]. Under regulation by autophagy-related genes (ATGs), double membranes enclose damaged or dysfunctional cytoplasmic components to form autophagosomes, which eventually fuse with lysosomes to form autolysosomes, achieving autophagic degradation [12, 13]. The basal level of autophagy is quite low but essential in the normal physiological state; however, the autophagy mechanism is rapidly up-regulated under starvation, hypoxia, and microbial invasion, including by bacteria and viruses [11, 14]. Activated autophagy functions to identify and eliminate pathogen-associated molecular patterns for cellular defence. Additionally, autophagy provides conditions such as substances and sites for viral replication and viral extracellular release, thus promoting persistent viral infection [15–17]. Regarding the pathogenesis of DHAV-1 [18], the diagnosis [19–22], immune molecular epidemiology [23, 24] and some gene functions [25] have been reported, though the interplay between autophagy and DHAV-1 infection remains unexplored.

In the present study, we sought to elucidate the effect of DHAV-1 infection on autophagy, to assess whether autophagy induction affects viral replication, and to determine the molecular mechanism by which DHAV-1 induces autophagy in duck embryo fibroblasts (DEFs). First, we monitored autophagosome formation following DHAV-1 infection and examined DHAV-1 genome copies and viral titres after pharmacological autophagy induction and inhibition. The structural proteins of DHAV-1 were then examined for autophagosomes, autophagolysosomes and PI3KC3 complexes. The results of this study confirm that autophagy activation in DEFs is dependent on DHAV-1 viral replication, and that autophagy activation can promote DHAV-1 proliferation and viral particle release from the intracellular to the extracellular space. Furthermore, the DHAV-1 structural protein VP1 interacts with the PI3KC3 protein to induce autophagy in DEFs through the PI3KC3 complex.

## Materials and methods

### Cells, virus strain, serum samples and plasmids

DEFs were extracted from specific pathogen-free 9- to 11-day-old duck embryos and cultured in Dulbecco’s Modified Eagle’s Medium (DMEM, 11995065, GIBCO) containing 10% foetal bovine serum (FBS, 10091148, GIBCO) to a cell density of approximately 90%. DHAV-1 strain H (GenBank Accession Number: JQ301467.1) is maintained in our laboratory, and its complete genome is available at GenBank. Duck anti-DHAV-1-VP3, rabbit anti-DHAV-1-VP1 or duck anti-DHAV-1 serum samples and DHAV-1 structural protein eukaryotic expression, GFP-LC3 and pmCherry-GFP-LC3 plasmids are also stored in our laboratory. To obtain a non-replicable DHAV-1 strain, DHAV-1 was inactivated at 100 °C for 1 h or exposed to UV light for 3 h with regular gentle shaking. Loss of infectivity was confirmed by detection of viral genome copies and titres.

### Virus infection and drug treatment

DHAV-1 was propagated in DEFs in serum-free DMEM for 2 h; the cells were then washed 3 times with phosphate-buffered saline (PBS, pH 7.4) and cultured in DMEM containing 2% FBS until harvest. For autophagy induction and inhibition assays, cells were pretreated with serum-free DMEM for 8 h, 100 nmol/L rapamycin (RAPA, V900930, Sigma) for 4 h or 50 μmol/L chloroquine (CQ, C6628, Sigma) for 24 h [26] or 3-methyladenine (3-MA, 110721, MedChemExpress) treatment for 12 h. An equal amount of DMEM was used as a control. Drug toxicity testing was performed using WST-1-Cell Proliferation and Cytotoxicity Assay Kit (C0035, Beyotime).

### Western blot analysis

To assess whether DHAV-1 infection and the DHAV-1 protein induce autophagy and autophagic flux in DEFs, we investigated expression of the autophagy marker protein LC3 and the autophagy substrate protein SQSTM1/p62 by western blotting. DHAV-1-infected target cells were washed 3 times with pre-chilled PBS and lysed with cell lysis buffer (P0013, Beyotime) and protease inhibitors (ST506, Beyotime). The supernatant was collected, and the protein concentration of the supernatant was determined using TaKaRa Bradford Protein Assay Kit (T9310A, Takara). Protein samples containing 2× loading buffer were boiled for 30 min, analysed by 15% sodium dodecyl sulfate–polyacrylamide gel electrophoresis (SDS–PAGE), and transferred to 0.22-µm nitrocellulose membranes. The membranes were blocked with 5% nonfat milk for 2.5 h at room temperature and incubated with primary antibodies, including rabbit anti-LC3B (L7543, Sigma), rabbit anti-SQSTM1/p62 (P0067, Sigma), mouse anti-Flag monoclonal (M185-3, MBL), mouse anti-β-actin (58169, CST), duck anti-DHAV-1-VP3, rabbit anti-DHAV-1-VP1 or duck anti-DHAV-1 antibodies, overnight at 4 °C. After washing 3 times with Tris-buffered saline and Tween (TBST) buffer for 1 h, the membrane was incubated with secondary antibodies, including goat anti-rabbit IgG (e62218, KPL), goat anti-mouse IgG (8890, CST) or goat anti-duck IgG (042506, KPL), for 2 h at room temperature. Detection was performed using HRP chemiluminescence (T7101A, Takara) and the Bio-Rad ChemiDoc MP system. ImageJ software (National Institutes of Health) was used to analyse and quantify the protein content.

### Transmission electron microscopy (TEM)

To observe the microstructure of autophagosomes, DEFs in 100-mm culture dishes were harvested at 48 h after DHAV-1 infection, and mock cells served as controls. The samples were harvested according to the processing method of Lilai Biotechnology for TEM. Images of ultrathin sections were observed and collected using an H-600IV TEM (Hitachi, Japan). The amount of GFP-LC3 puncta among 50 cells was counted for each group.

### Fluorescence microscopy

To observe formation of autophagosomes and autolysosomes, the GFP-LC3 or pmCherry-GFP-LC3 plasmid was transfected into DEFs using Lipofectamine™ 2000 Transfection Reagent (11668019, Thermo Fisher Scientific) for 24 h followed by treatment with DHAV-1 or the DHAV-1 structural protein or RAPA. Mock-infected cells were used as controls. The cell samples were fixed with 4% paraformaldehyde for 30 min at 4 °C and then stained with 2-(4-amidinophenyl)-6-indolecarbamidine dihydrochloride (DAPI, C1002, Beyotime) for 30 min at 37 °C. LC3 puncta were visualized under a Carl Zeiss confocal microscope (Axio Imager A2, Germany).

### Real-time quantitative PCR (RT–qPCR)

Specific primers for the GenBank database DEF gene ATG5 (NW_004678004.1) were designed by Primer Premier 5.0; the upstream primer is 5′-GAAGCCGAGCCTTACTATTT-3′, and the downstream primer is 5′-AAACCAATCGGGTAATGCCA-3′. Primers for qRT-PCR detection of virus copy number and the internal reference gene of DEFs are as described in the reference [20]. RNA was extracted using RNAiso Plus (9109, Takara) and reverse transcribed using the PrimeScript™ RT kit with gDNA Eraser (RR047A, Takara). The target gene was amplified by RT–qPCR using the above specific primers.

To examine the effect of DHAV-1 infection on ATG5 transcript levels, DEFs were treated with starvation (medium without FBS), CQ, or DHAV-1, as described above. Cell samples were harvested and ATG5 transcript levels were detected using relative RT-qPCR. To examine the effect of autophagy on DHAV-1 proliferation, DEFs were treated with RAPA, CQ, or equivalent medium, as described, before DHAV-1 infection. RNA was extracted and reverse-transcribed by RT–qPCR for detection of viral genome copies. RT–qPCR was performed using CFX ConnectTM Optical Module Real-Time PCR System (Bio-Rad, USA).

### Tissue culture infective dose

The effect of autophagy induction on DHAV-1 proliferation was measured by using the TCID_50_ method. In a 96-well plate (AB0751, Thermo Fisher Scientific), DEFs were infected with serially diluted DHAV-1 from 10^–1^ to 10^–10^ and cultured at 37 °C and 5% CO_2_, and lesions were observed continuously. Eight replicates were established for each dilution. Viral titres were calculated according to the Reed-Muench method.

### Coimmunoprecipitation (Co-IP) analysis

For Co-IP assays, cell lysates were incubated with anti-HA-Tag or anti-Flag-Tag mAb overnight in a rolling incubator at 4 °C. The mixture was then incubated with protein A + G agarose gel (purchased from Bio-Rad) at 4 °C for 4 h, washed 5 times with 1% Triton PBS buffer, and subjected to immunoblot analysis.

### Statistical analysis

Statistical data are presented as the mean ± standard deviation (SD). Three biological replicates were performed for each experiment. Differences between two groups were analysed using Student’s *t* test, and results are presented as *P* < 0.05 (*), *P* < 0.01 (**) or *P* < 0.001 (***); “ns” indicates no significant difference.

## Results

### Autophagy is induced by DHAV-1 infection in DEFs

**Figure 1:**
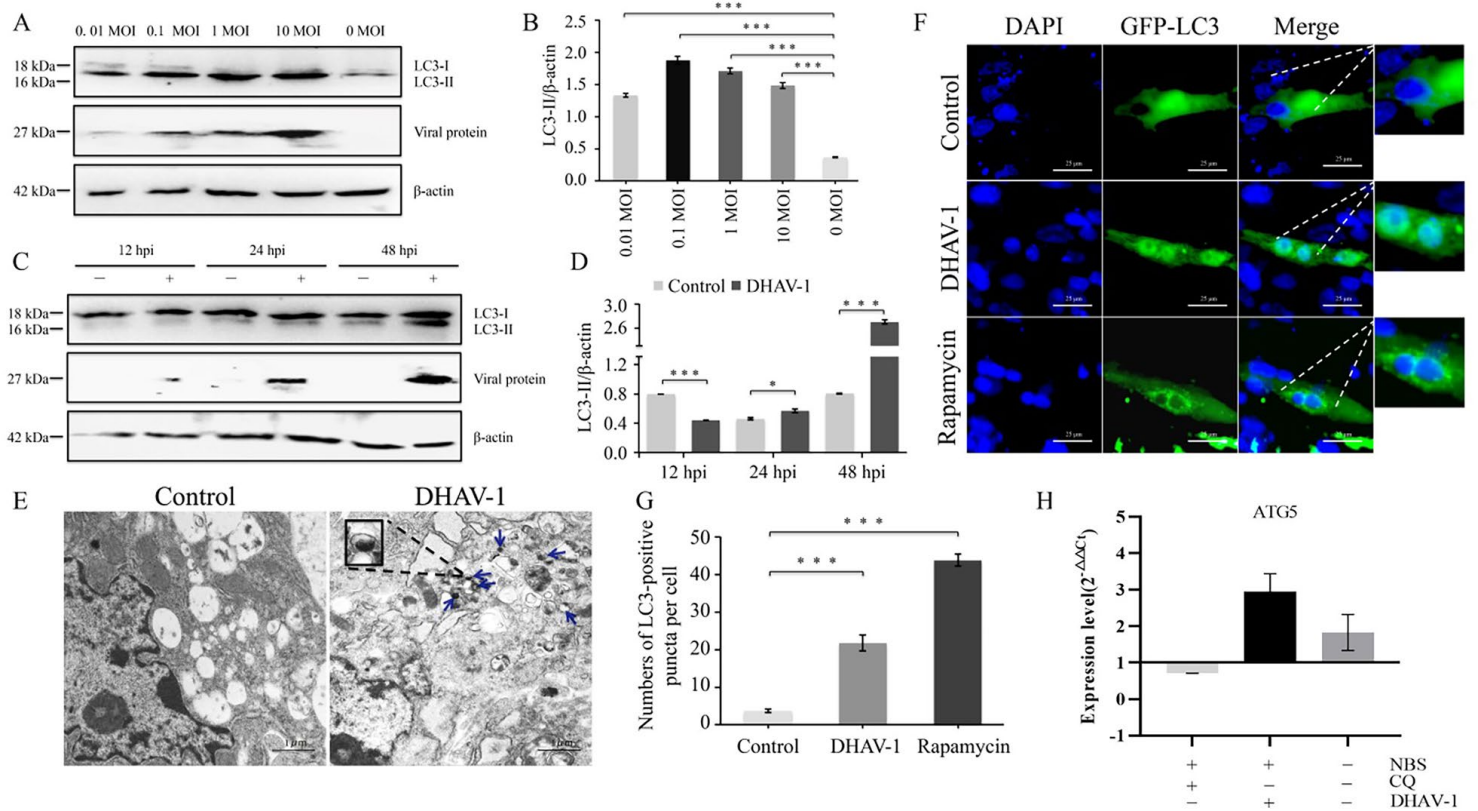
**DHAV-1 infection induced autophagy in DEFs.** **A** DEFs were or were not infected with 0.01–10 MOI DHAV-1, as described in Materials and methods. Samples were harvested from three biological replicates at the indicated times, and LC3, VP3 and β-actin protein expression was detected by western blot with specific antibodies. **B** Relative expression levels of targeted proteins were quantified and analysed. Data represent the mean ± SD of three independent experiments. Differences between two groups were analysed using Student’s *t* test and are presented as *P* < 0.05 (*), *P* < 0.01 (**) and *P* < 0.001 (***). **C** DEFs were or were not infected with 1 MOI DHAV-1 for 12 h, 24 h or 48 h and harvested to detect expression of targeted proteins by western blot with specific antibodies. Three biological replicates were performed and tested. **D** Protein expression ratios of LC3-II/β-actin were quantified and analysed. Data represent the mean ± SD of three independent experiments. Differences between two groups were analysed using Student’s *t* test and are presented as *P* < 0.05 (*) and *P* < 0.001 (***). **E** DEFs were or were not infected with 1 MOI DHAV-1 for 48 h and observed by TEM. The blue arrows indicate single- or double-membrane autophagosome-like vesicles. **F** DEFs transfected with GFP-LC3 plasmid were mock infected or infected with DHAV-1 in the absence or presence of 100 nmol/L RAPA, and GFP-LC3 puncta were observed by fluorescence microscopy. Blue, DAPI staining of nuclei; green, GFP-LC3 puncta. Scale bar, 25 μm. **G** The number of LC3-positive puncta per cell was calculated and is presented as the mean ± SD of three replicates per group. Differences between two groups were analysed using Student’s *t* test and are presented as *P* < 0.001 (***). **H** The relative transcription level of the ATG5 gene was detected by RT–qPCR in the absence or presence of NBS, CQ (50 μmol/L) or DHAV-1 (MOI = 1) for the indicated times, with three replicates per group. RAPA, rapamycin; CQ, chloroquine.

Microtubule-associated protein 1 light chain 3 (LC3; also known as MAP1LC3) is an autophagy marker that exists in two forms: cytoplasmic LC3-I and membrane-bound LC3-II [16, 27, 28]. Under ATGs regulation, LC3 precursors are first processed into LC3-I with exposed glycine residues at the carboxyl end, which bind to LC3-II with phosphatidylethanolamine [29]. Therefore, LC3-II anchored at the autophagosome membrane is an index of autophagy. After culturing DEFs with DHAV-1 at various multiplicities of infection (MOIs) for a given period of time, lipidation of the LC3 protein and expression of the DHAV-1 VP3 protein were detected by western blot using appropriate antibodies. The VP3 protein results suggested that DHAV-1 infection was successful and proportional to the amount of virus added. The LC3 protein results suggested that LC3-I could be induced to become lipidated to LC3-II; the greatest effect was observed at 1 MOI of DHAV-1 (Figure 1A, B). The relative expression level of LC3-II in the DHAV-1 infection group was much higher than that in the control group at 48 h after infection, but there was no significant difference between these groups at the other designated times (Figure 1C, D). The infection condition of 1 MOI DHAV-1 for 48 h was used for subsequent experiments. Single- and double-membrane autophagosome-like vesicles were observed in DHAV-1-infected DEFs by TEM, whereas mock-infected cells showed no such cytoplasmic vesicles (Figure 1E). To verify autophagy activation in DHAV-1-infected cells, the enhanced green fluorescent protein (EGFP)-LC3 plasmid was transfected into DEFs infected with DHAV-1, with uninfected cells used as controls, and the dot formation of the GFP-LC3 fusion protein was investigated. Unlike the GFP-LC3 distribution in control cells, the GFP-LC3 protein in the DHAV-1-infected cells aggregated into bright dots (Figure 1F, G), indicating that DHAV-1 infection induced formation of autophagosomes.

### Pharmacological inhibition of autophagy restrains DHAV-1 proliferation and viral release

**Figure 2:**
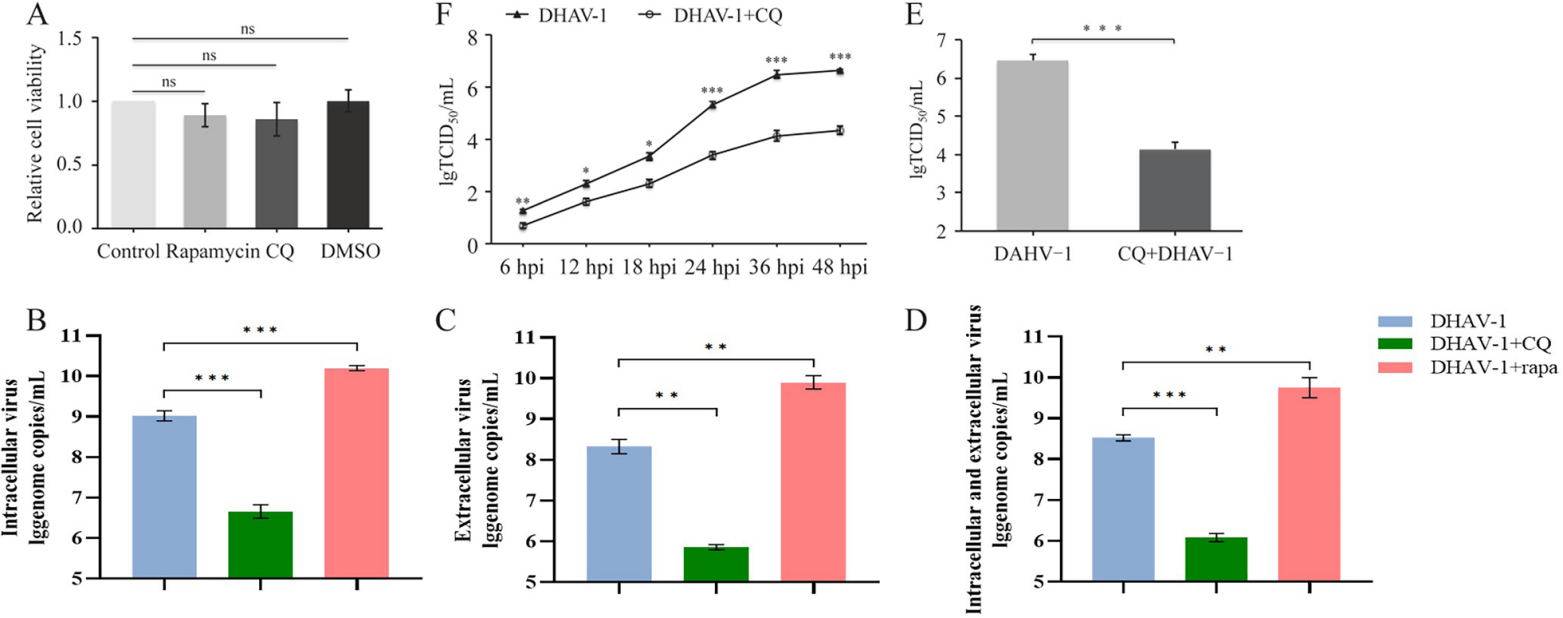
**Pharmacological inhibition of autophagy restrained DHAV-1 proliferation and viral release.** **A** Pharmacology did not affect cell viability. After DEFs were treated with 100 nmol/L RAPA, 50 μmol/L CQ or the solvent DMSO, as described in Materials and methods, the cell viability of each group was assessed by the WST-1 assay. The optical density of sample wells was determined at 450 nm (OD_450_) and normalized to the optical density of the blank well. Data shown represent the mean ± SD of three independent experiments. “ns” indicates no significant difference, *P* > 0.05. **B**–**D** After pretreatment with CQ (50 μmol/L) or RAPA (100 nmol/L) or no pretreatment, DEFs were infected with DHAV-1, and the number of extracellular and intracellular DHAV-1 viral copies was detected by RT–qPCR. All data were derived from three separate sets of experiments. **E**, **F** DEFs were treated with 50 μmol/L CQ before DHAV-1 infection, the cells were observed, and cytotoxic effects were recorded. Viral titres were calculated and are expressed as TCID_50_ per mL. Differences between two groups were analysed using Student’s t test and are presented as *P* < 0.05 (*), *P* < 0.01 (**) and *P* < 0.001 (***).

The effects of the drugs used in this study on cell viability were detected by the WST-1 assay. The viability of DEFs treated with drugs and drug solvents was not significantly different from that of the mock-treated cells (Figure 2A), suggesting that the pharmacological treatment did not affect cell viability. Autophagic cells can produce extracellular microvesicles (EMVs) through the action of ATG5 and provide sites for replication of picornavirus and related proteins, thus promoting viral replication and proliferation. To explore the relationship between autophagy and DHAV-1 replication, an RT–qPCR standard curve of the DHAV-1 structural protein VP0 was established to detect the effects of an autophagy inhibitor and an agonist on the number of DHAV-1 viral copies. Compared with DHAV-1 infection alone, RAPA treatment increased the number of intracellular and extracellular viral copies, though CQ treatment significantly reduced the number (Figure 2B, C), suggesting that autophagy induction promotes DHAV-1 replication and that autophagy inhibition decreases DHAV-1 replication. Additionally, autophagy promoted DHAV-1 release, as evidenced by the contrasting numbers of intracellular and extracellular viral proteins in each group (Figure 2B–D). When CQ-treated or untreated DEFs were cultured for 6 h, 12 h, 18 h, 24 h, 36 h, or 48 h, the viral titres of the CQ-treated group were always lower than those of the untreated group, as determined by the TCID_50_ method. The numerical difference was related to the length of DHAV-1 infection and was greatest at 36 h after DHAV-1 infection (Figure 2E, F).

### A DHAV-1 structural protein promotes induction of autophagy but not intact autophagic flux in DEFs

**Figure 3:**
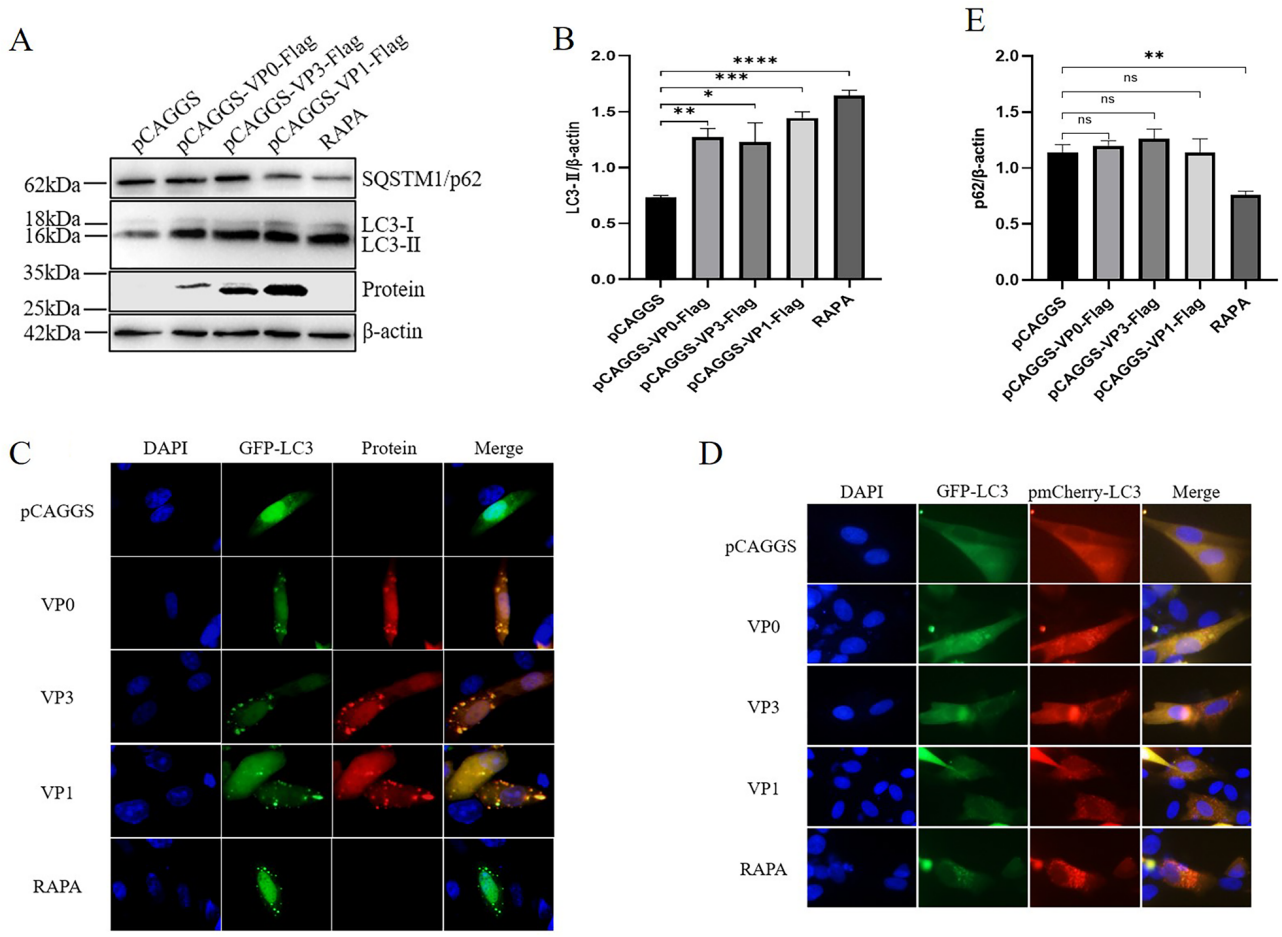
**The DHAV-1 structural protein promotes induction of autophagy but not intact autophagic flux in DEFs.** **A** VP0, VP3 and VP1 proteins were expressed in DEFs, and expression of the SQSTM1/p62 protein and transformation of the LC3 protein were detected by western blot. **B** Grey value analysis of changes in LC3 conversion levels after VP0, VP3 and VP1 proteins were expressed in DEFs, *P < 0.05, **P < 0.01, ***P < 0.001, and ****P < 0.0001. **C** GFP-LC3 was co-transfected with the VP0, VP3 and VP1 protein plasmids into DEFs. After samples were collected and fixed, protein expression levels of VP0, VP3 and VP1 were detected by labelling antibodies, and punctate aggregation of LC3 was observed by microscopy. **D** Using the pmCherry-GFP-LC3 dual-fluorescence system, the VP0, VP3 and VP1 proteins were expressed in DEFs, and green fluorescence and red fluorescence were observed under a microscope. **E** Grey value analysis of changes in SQSTM1/p62 protein levels after expression of VP0, VP3 and VP1 proteins in DEFs; ns indicates no difference, **P < 0.01.

It has been demonstrated that DHAV-1 can induce autophagy in DEFs. To further explore the molecular mechanism by which autophagy is induced in DEFs, we evaluated the DHAV-1 structural protein. First, conversion of LC3 was detected by western blot, and the structural proteins VP0, VP1 and VP3 of DHAV-1 promoted LC3 conversion from LC3-I to LC3-II (Figure 3A, B). The results of IFA experiments showed that VP0, VP1 and VP3 promote GFP-LC3, with obvious punctate aggregation (Figure 3C), indicating that DHAV-1 structural proteins induce autophagy in DEFs.

Autophagy flux is a dynamic and continuous process that involves formation of autophagosomes, transfer of autophagy substrates to lysosomes and substrate degradation in lysosomes in vivo [26, 30]. Both autophagy activation and autolysosome formation inhibition can result in autophagosome accumulation, and we detected autophagic flux to explore the cause of autophagy in DHAV-1-infected DEFs.

The dual fluorescence system pmCherry-GFP-LC3 is a more reliable method for quantitative autophagy analysis than the GFP-LC3 system. EGFP is sensitive to a low-pH environment, whereas the pmCherry protein is resistant to an acidic environment. When autophagy is induced, pmCherry-GFP-LC3 is expressed in cells anchored to autolysosomes, and EGFP is quenched; thus, yellow-labelled autophagosomes and red-labelled autolysosomes are observed [30, 31]. The number of these yellow and red spots increases when autophagic flux increases. Samples were collected for indirect immunofluorescence experiments at 36 h after co-transfection with pmCherry-GFP-LC3 and DHAV-1 structural protein plasmids. Microscopy observation showed that RAPA promoted LC3 punctate aggregation and more red fluorescence; however, the DHAV-1 structural protein promoted LC3 punctate aggregation but more yellow fluorescence than red fluorescence (Figure 3D). This indicates that the DHAV-1 structural protein may not promote complete autophagic flow in DEFs.

The SQSTM1/p62 protein, an autophagy degradation substrate, targets ubiquitinated substrates to autophagosomes via LC3, resulting in autolysosome-mediated degradation of SQSTM1/p62 proteins and ubiquitinated substrates and maintaining cellular homeostasis [32]. Therefore, expression of SQSTM1/p62 protein indicates autophagy flux. The DHAV-1 structural protein was transfected into DEFs; RAPA treatment and pCAGGS transfection were used as controls. The expression level of SQSTM1/p62 protein was then determined by western blot (Figure 3A, E). RAPA treatment down-regulated SQSTM1/p62 protein expression. However, transfection of pCAGGS with DHAV-1 structural protein led to no significant changes in the SQSTM1/p62 protein. These results suggest that the DHAV-1 structural protein has no effect on the level of autophagic flux in DEFs.

### The DHAV-1 VP1 protein induces autophagy in DEFs via the PI3KC3 complex

**Figure 4:**
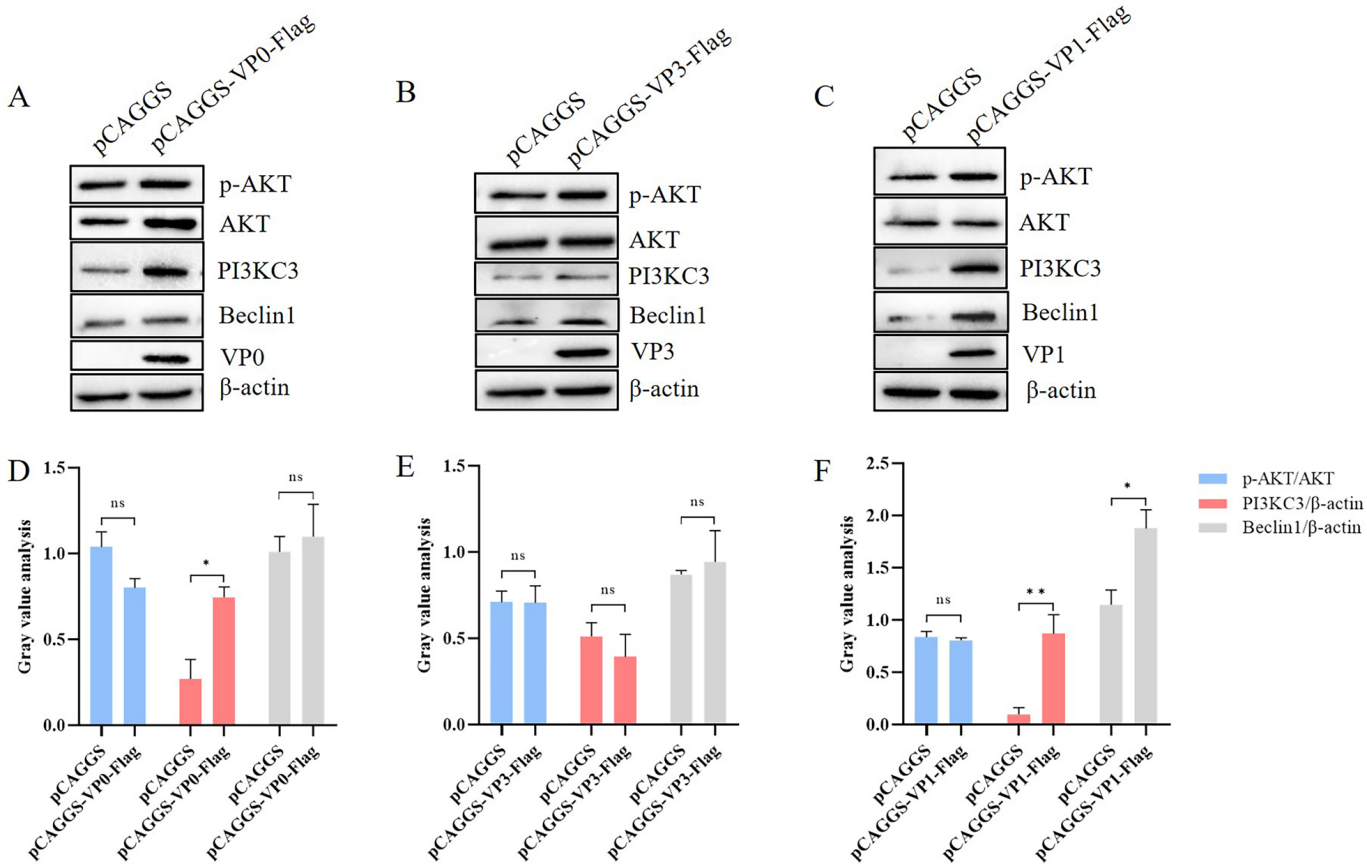
**The DHAV-1 VP1 protein induces autophagy in DEFs via the PI3KC3 complex.** **A**–**C** VP0, VP3 and VP1 protein plasmids were transfected into DEFs, and protein expression levels of p-AKT, AKT, PI3KC3 and Beclin1 were detected by western blot. **D**–**F** Grey value analysis of western blot results of p-AKT, AKT, PI3KC3 and Beclin1 in DEFs after transfection of VP0, VP3 and VP1 protein plasmids; ns indicates no significant difference, *P < 0.05, **P < 0.01.

The PI3KC3 complex, which is mainly composed of Vps34, p150 and Beclin1 proteins, is crucial for regulation of autophagy [33, 34]. Hence, we examined the effects of DHAV-1 structural proteins on expression of PI3KC3 and Beclin1 proteins and observed that VP3 protein had no significant effect (Figure 4B, E), indicating that VP3 induces autophagy in DEFs without PI3KC3 complex involvement. Although the VP0 protein increased expression of PI3KC3, it had no effect on Beclin1 (Figure 4A, D), indicating that VP0 induces autophagy in DEFs independent of the PI3KC3 complex. VP1 also increased expression levels of PI3KC3 and Beclin1 proteins (Figure 4C, F), indicating that VP1 induces autophagy in DEFs through the PI3KC3 complex.

### The VP1 protein interacts with the PI3KC3 protein

**Figure 5:**
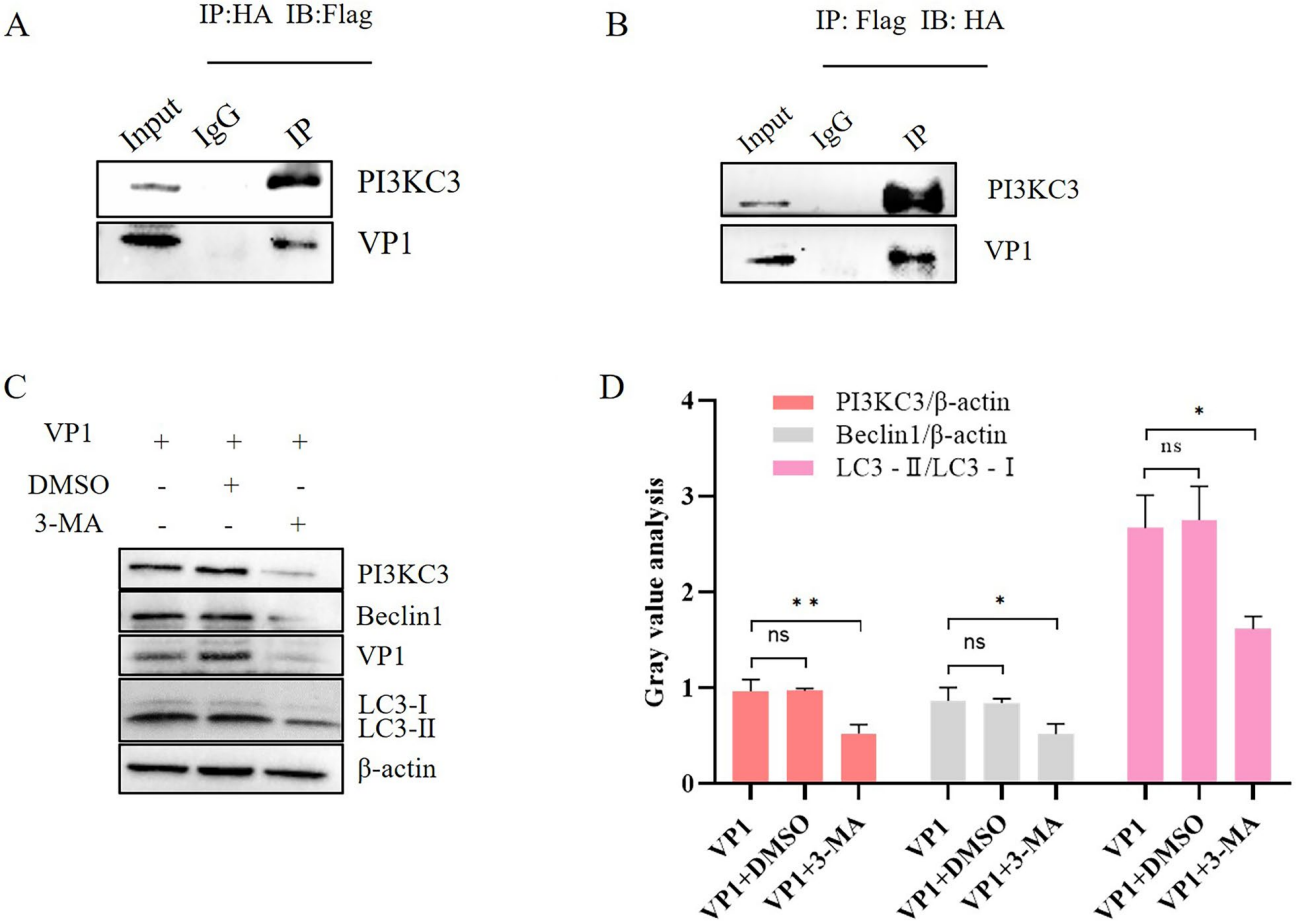
**The VP1 protein interacts with the PI3KC3 protein.** **A** VP1-Flag and PI3KC3-HA plasmids were co-transfected into DEFs, cell lysates were subjected to Co-IP assays with a Flag-tagged antibody or normal IgG as negative controls, and co-immunoprecipitated proteins were detected by western blot analysis. **B** VP1-Flag and PI3KC3-HA plasmids were co-transfected into DEFs, cell lysates were subjected to a Co-IP assay with an HA-labelled antibody or normal IgG as negative controls, and co-immunoprecipitated proteins were detected by western blot analysis. **C** DEFs were treated with 3-MA, and transformation of the PI3KC3 complex and LC3 were detected by western blotting. **D** Grey value analysis of transformation changes of the PI3KC3 complex and LC3 after 3-MA drug treatment; ns indicates no difference, *P < 0.05, **P < 0.01.

DHAV infection up-regulates PI3KC3 protein expression, but its molecular mechanism has not been reported [35]. As our experiments demonstrate that the VP1 protein induces autophagy through the PI3KC3 complex, we constructed a pCAGGS-PI3KC3-HA eukaryotic expression plasmid and explored the interaction between VP1 protein and PI3KC3 protein by Co-IP experiments. The results showed an interaction between these proteins (Figure 5A, B).

To further study the effect of PI3KC3 protein on VP1 protein-induced autophagy in DEFs, cells were treated with the PI3KC3 protein inhibitor 3-MA drug and transfected with a VP1 protein plasmid. Western blot assays were used to detect transformation of the PI3KC3 protein, Beclin1 protein and LC3. The results showed that 3-MA treatment significantly reduced expression of PI3KC3 and Beclin1; transformation of LC3 was also inhibited (Figure 5C, D), indicating that 3-MA-mediated knockdown of PI3KC3 inhibits VP1 protein-induced DEF self-regulation.

## Discussion

Autophagy is a highly conserved eukaryotic biological process that maintains cellular homeostasis through self-repair and renewal; as a host defence mechanism, autophagy promotes resistance to pathogen infection [36–38]. On the other hand, autophagy provides pathogen replication sites, materials and release pathways to promote pathogen proliferation as well as persistent infection [39, 40]. DHAV-1 is one of the most common highly lethal hepatitis pathogens in ducklings, with serious consequences [3]. However, the molecular mechanistic relationship between autophagy and DHAV-1 infection has remained unclear. In this study, we first demonstrated that DHAV-1 infection induces autophagy in DEFs and that pharmacological inhibition of autophagy inhibits DHAV-1 proliferation and extracellular release. We then showed that the DHAV-1 structural protein can induce autophagy in DEFs, and it was found that VP1 affects the PI3KC3 complex by interacting with the PI3KC3 protein, thereby inducing autophagy in DEFs.

Within a certain range, autophagy induction is directly proportional to the dose and duration of viral infection [41, 42]. For instance, autophagy in DEFs is consistently enhanced with 0.1–10 MOI of duck plague virus (DPV). Intracellular vesicle acidification promotes maturation of infectious poliovirus particles. The gene ICP34.5 of herpes simplex virus 1 inhibits autophagy in host cells, whereas autophagy is activated with high-dose virus infection [43, 44]. In this study, the level of autophagy continued to increase in DEFs within the range of DHAV-1 MOI 0.01–1, but the degree of increase in autophagy was reduced at 10 MOI. Autophagy induction also correlates with viral infection time. Autophagy induced by DPV infection showed a change with prolonged infection, gradually increasing from 12 to 48 h post infection but decreasing from 48 to 72 h [26]. Diverse picornaviruses, including foot-and-mouth disease virus (FMDV), invade by specifically recognizing and binding with cell surface receptors and rely on the ATG5 pathway to induce autophagy at the early stage of infection [45]. Our study showed that the transcriptional level of the ATG5 gene was increased during DHAV-1 infection, indicating high levels of autophagy. As anti-LC3 antibodies recognize LC3-II more efficiently than LC3-I, LC3-II expression values are often used to indicate autophagy levels after Western blot scanning and band quantification [46, 47]. LC3 became lipidated from LC3-I to LC3-II at 48 h after DHAV-1 infection compared to the preinfection period. In the presence of DHAV-1 infection, fluorescently labelled GFP-LC3 proteins aggregated as bright spots. During this process, the autophagosome precursor continues to extend, wrapping cytoplasmic components in the membrane to form a closed spherical structure called the autophagosome [32]. In this study, single- and double-membrane autophagosome vesicles were observed in DHAV-1-infected DEFs. In conclusion, DHAV-1 infection can induce autophagy in DEFs.

Viral infection in host cells comprises three stages: the infection stage of adsorption and penetration; the proliferation stage of uncoating and biosynthesis; and the mature stage of assembly and release [48, 49]. Identifying the stage at which viral infection induces autophagy has long been a hot research topic. High-temperature treatment and UV irradiation are the two most common methods to inactivate pathogens [50, 51]. A series of studies determined that heat-inactivated virus cannot induce autophagy and concluded that autophagy induction depends on viral replication [12, 17, 52]. However, these studies did not involve any experiments with UV-inactivated virus [44, 53]. In this study, UV-inactivated DHAV-1 still induced autophagy in host cells, though heat-inactivated DHAV-1 did not, and the structure and function of DHAV-1 viral proteins was thus associated with autophagy induction rather than viral activity. The causes and mechanisms need to be further explored.

The role of autophagy in viral replication has always been controversial. In addition to its role as a natural barrier against pathogenic infection, autophagy benefits viral replication, proliferation and extracellular release [54–58]. Our results showed that an autophagy inducer could increase the number of extracellular and intracellular viral genome copies. Cell lysis is a traditional viral release pathway. However, autophagosome-mediated exit without lysis represents a new release pathway for enteroviruses such as PV and CV [39, 49, 58]. Compared with the control group and DHAV-1-infected group, RAPA treatment resulted in the largest differences in intracellular and extracellular viral genome copies and the closest extracellular and the total cellular (intracellular plus extracellular) genome copies; thus, autophagy promotes intracellular to extracellular viral particle release. Berryman et al. [12] reported that inhibition of autophagy caused by knocking down the ATG5 gene restrains FMDV replication. In our study, we blocked autophagy via CQ treatment, which inhibited fusion of autophagosomes and lysosomes, after which we then infected cells with replication-competent DHAV-1. The TCID_50_ results showed that viral titres were significantly decreased by CQ treatment, indicating that autophagy inhibition indeed restrains DHAV-1 proliferation. These results suggest that autophagy induction benefits DHAV-1 proliferation and extracellular release.

The structural proteins of picornaviruses can activate autophagy rapidly; the VP1 protein is currently the most widely reported such protein. VP1 of picornaviruses induces LC3-I lipidation to LC3-II and thus colocalizes with LC3-II in autophagosomes, suggesting that the VP1 protein induces autophagy in host cells such as EV71 [15, 59]. In this study, expression of the DHAV-1 structural proteins VP0, VP1 and VP3 increased LC3 lipidation and induced autophagosome formation. When determining the cause of autophagosome accumulation, autophagy flux is a more accurate indicator than autophagosome detection. Among tracer proteins, GFP is very sensitive, and when autolysosomes form, the internal acidity quenches the green fluorescence of GFP. Our results indicate that the fluorescence of the pmCherry protein remained unchanged but that GFP was quenched in DEFs transfected with the pmCherry-GFP-LC3 plasmid. In this study, the DHAV-1 structural protein and pmCherry-GFP-LC3 plasmids were co-transfected. Microscopic observation showed that GFP expressed with the DHAV-1 structural protein was not significantly quenched, indicating that autophagy–lysosome formation was blocked. During autolysosomal degradation, the SQSTM1/p62 protein targets ubiquitinated substrates to autophagosomes through LC3, ultimately leading to degradation of SQSTM1/p62 and ubiquitinated substrates in autophagosomes, thereby maintaining the intracellular steady state [32]. Therefore, the level of SQSTM1/p62 protein expression can be used to monitor autophagic flux. We found that the DHAV-1 structural protein blocks degradation of SQSTM1/p62. These results suggest that the DHAV-1 structural protein induces autophagy but not full autophagic flux in DEFs.

The PI3KC3 family is divided into three categories, among which the class I lipid kinase class (PI3KC1) is the upstream activator of protein kinase B (Akt) and forms PI3K/AKT/mTOR with the downstream gene mTOR of AKT to inhibit autophagy [60]. Class III lipid kinase (PI3KC3, also known as VPS34) forms a PI3KC3 complex with the autophagy-related protein Beclin-1, which is essential for autophagosome formation [61]. 3-MA has been used in the study of autophagy as a broad inhibitor of PI3KC3. In this study, AKT, PI3KC3 and Beclin1 were detected, and the DHAV-1 structural protein did not affect the protein level or phosphorylation level of AKT, indicating that it induces autophagy in DEFs without AKT-related signalling. The structural protein VP1 of DHAV-1 promotes expression of PI3KC3 and Beclin1 proteins, whereas the structural proteins VP0 and VP3 do not affect the PI3KC3 complex, indicating that VP1 induces autophagy through the PI3KC3 complex but that VP0 and VP3 do not. The complex induces autophagy in DEFs, and we continued to explore the molecular mechanism of VP1 protein-induced autophagy in DEFs. Co-IP experiments showed that the VP1 protein interacts with the PI3KC3 protein. Using 3-MA treatment, VP1-induced LC3 conversion was also blocked after PI3KC3 was inhibited. These results suggest that the host protein PI3KC3 interacts with the VP1 protein and promotes VP1 protein-induced autophagy in DEFs.

In conclusion, this study demonstrates that autophagy induction is dependent on the integrity of the DHAV-1 viral protein and promotes the proliferation and extracellular release of DHAV-1 in DEFs. It was also found that the DHAV-1 structural protein VP1 induces autophagy in DEFs by interacting with the host protein PI3KC3 to regulate the PI3KC3 complex. This study provides a new perspective for basic research materials on DHAV-1-host interactions and antiviral strategies and drug control.
